# Awareness, knowledge, and practices related to hepatitis B and hepatitis C in the Republic of Uzbekistan—Results from a population-based survey, 2022

**DOI:** 10.1186/s12889-025-25990-1

**Published:** 2026-01-23

**Authors:** Rania A. Tohme, Shaun Shadaker, Elizaveta Joldasova, Aybek Khodiev, Dilafkor Mirdjalilov, Zulfiya Abdurakhimova, Botirjon Kurbanov, Nino Khetsuriani, Shakhida Karamatova, Erkin Musabaev

**Affiliations:** 1 Division of Viral Hepatitis, U.S. Centers for Disease Control and Prevention (CDC), Atlanta, GA USA; 2 Research Institute of Virology of the Republican specialized scientific and practical medical center of epidemiology, microbiology, infectious and parasitic diseases, Tashkent, Uzbekistan; 3 Integral Global Health, Tashkent, Uzbekistan; 4 Division of Global Health Protection, CDC Uzbekistan Country Office, Tashkent, Uzbekistan; 5 Committee for Sanitary and Epidemiological Welfare and Public Health, Ministry of Health of the Republic of Uzbekistan, Tashkent, Uzbekistan

**Keywords:** Awareness, Knowledge, Hepatitis b, Hepatitis c, Uzbekistan

## Abstract

**Background:**

Patient and healthcare provider knowledge were previously found to be significantly associated with viral hepatitis testing in Uzbekistan. However, no survey has assessed awareness and knowledge of viral hepatitis among the general population.

**Methods:**

In 2022, we conducted a cross-sectional population-based survey among persons aged≥18 years in seven of Uzbekistan’s 14 regions representing 60% of the population. We conducted face-to-face interviews to assess hepatitis B and C awareness, knowledge, and related practices. Knowledge scores for hepatitis B and C were calculated based on the total number of correct answers. Adjusted odds ratios (aOR) and 95% confidence intervals (CI) were computed to assess factors associated with awareness and knowledge scores. Analyses were weighted.

**Results:**

Of 10,000 targeted adults, 9,066 (91%) participated in the survey. Overall, 54.7% (95% CI: 46.4–62.8) and 39.0% (95% CI: 33.0–45.4) were aware of hepatitis B and hepatitis C, respectively. Females compared to males (hepatitis B: aOR: 2.61 (95% CI: 1.91–3.56); hepatitis C: 2.15 (1.76–2.63)) and persons with higher education compared to those with technical education (hepatitis B: aOR: 1.89 (95% CI:1.32–2.71); hepatitis C: 2.30 (1.63–3.25)) had higher hepatitis B and hepatitis C awareness. The median knowledge scores for hepatitis B and hepatitis C transmission were 4.9 (IQR: 4.2–6.3) out of nine and 4.8 (IQR: 4.2–5.9) out of eight, respectively. Median knowledge scores for prevention methods were 1.7 (IQR: 1.2–2.4) and 2.3 (IQR: 0.7–2.9) out of five for hepatitis B and hepatitis C, respectively. Persons ≥50 years old compared to 18–29 years old and those with less than secondary education compared to those with technical education had lower knowledge scores for hepatitis B. Living outside of Tashkent city and being unemployed were associated with having lower knowledge scores for hepatitis C. Overall,28.8% reported having ever been tested for hepatitis B and 17.7% for hepatitis C; while 8.2% and 2.3% reported being told that they have hepatitis B and hepatitis C, respectively.

**Conclusions:**

Educational campaigns are needed to promote population awareness and address knowledge gaps to increase uptake of interventions aimed at eliminating viral hepatitis in Uzbekistan.

**Supplementary Information:**

The online version contains supplementary material available at 10.1186/s12889-025-25990-1.

## Background

Hepatitis B and hepatitis C impact over 300 million persons worldwide and cause almost 1.3 million deaths every year resulting from cirrhosis and liver cancer [1]. In 2016, the World Health Assembly adopted a resolution to eliminate viral hepatitis as a public health problem aiming to reduce new infections by 90% and mortality by 65% by 2030 [2]. Countries in Eastern Europe and Central Asia have the highest prevalence of hepatitis B virus (HBV) and hepatitis C virus (HCV) infections in the European region. There are no accurate nationwide estimates for the prevalence of viral hepatitis in Uzbekistan; various modeling estimates report a prevalence of chronic hepatitis B ranging from 2.1% to 4.3% and a prevalence of chronic hepatitis C ranging from 3.0% to 3.7% [3–6].

In 2017, a Resolution by the Cabinet of Ministers in Uzbekistan No. 537 approved a set of measures to further improve diagnosis, prevention, and treatment of infectious diseases, including viral hepatitis during 2017–2021. As a result, Uzbekistan substantially stepped up its efforts against hepatitis B and hepatitis C. During 2019–2021, simplified test-and-treat strategies for hepatitis B and hepatitis C were implemented in primary healthcare facilities starting in Tashkent city first and then expanded to seven regions [7]– [8]. In 2022, the president of Uzbekistan signed a resolution to counter the spread of viral hepatitis which includes, among other measures, providing free hepatitis B and hepatitis C screening for the population, free treatment for hepatitis C for those infected, and free hepatitis B vaccination for healthcare workers in all regions [9].

Despite government support and commitment to the elimination of viral hepatitis in Uzbekistan and availability of free screening and treatment, adherence to hepatitis B and hepatitis C care and treatment has been reported to be low in Uzbekistan. During 2019–2020, 13–14% of persons diagnosed with hepatitis B or hepatitis C were lost to follow up after the first screening and an additional 62–66% did not seek further care and treatment [7]. In 2022, a qualitative study assessed the barriers and facilitators to viral hepatitis testing in Uzbekistan among a range of stakeholders including the general population [10]. Identified barriers to viral hepatitis testing included lack of awareness and knowledge about viral hepatitis among healthcare providers and the general population, low health literacy, limited awareness of locations and costs of testing, lack of time to get tested, and fear of stigma [10].

No survey has previously assessed awareness and knowledge of viral hepatitis among the general population in Uzbekistan. Such information is needed to develop tailored educational campaigns that would reach all socio-demographic groups. Therefore, this study was conducted to assess awareness, knowledge and practices related to hepatitis B and hepatitis C in Uzbekistan in order to provide evidence-based information that would guide the design and dissemination of educational material on hepatitis B and hepatitis C for the general population.

## Methods

### Study population and sampling methods

**Fig. 1:**
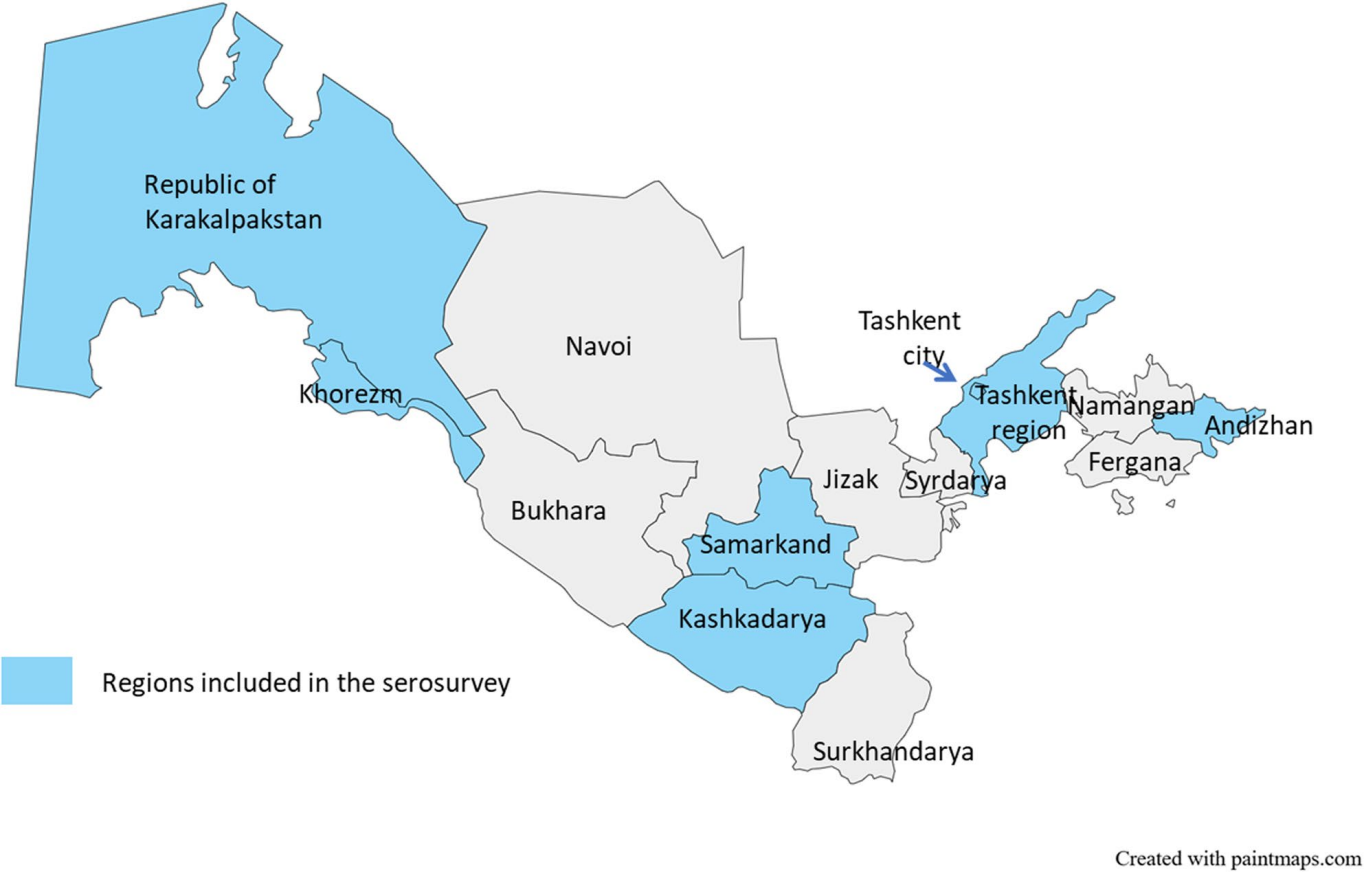
Map of Uzbekistan showing the regions selected for the survey

In 2022, we conducted a two-stage cluster sampling cross-sectional survey targeting adults (≥ 18 years of age) living in seven of Uzbekistan’s 14 regions representing 58% of the country’s population. The selected regions (1st level administrative areas) were Andijan, Samarkand, Kashkadarya, Khorezm, the Republic of Karakalpakstan, Tashkent, and Tashkent City (Fig. 1). The knowledge, awareness, and practices survey questions were incorporated into a serological survey for SARS-CoV2 and viral hepatitis. Selection of those regions was made to include regions with low, middle and high prevalence of SARS-CoV-2. A sample size of 10,000 adults was needed for an estimated prevalence of 25% for anti-SARS-CoV-2 with a precision of 1% at the 95% confidence interval, a design effect of 2, and an anticipated 5% non-response rate. This sample size was sufficient to assess awareness, knowledge and practices related to hepatitis B and hepatitis C (5,300 study participants were estimated to be needed if we consider that 50% of the study population were aware of hepatitis B or hepatitis C with a precision of 2% at the 95% confidence interval, a design effect of 2, and an anticipated 10% non-response rate).

In the first stage, primary healthcare clinics (PHCs) served as clusters. PHC clinics are assigned to a catchment area and all residents living in that catchment area are registered to receive health services at their respective PHC clinic. A total of 100 primary healthcare (PHC) clinics were randomly selected in the seven selected regions and allocated proportionate to population size and urban/rural population proportion in each region. In each selected PHC clinic, 100 adults were randomly selected from a list of the population served by this clinic. Participants were enrolled in the survey after providing informed consent. This survey excluded institutionalized individuals such as incarcerated people.

### Data collection

Data collection was conducted face-to-face by trained physicians using a structured paper-based questionnaire during March–July 2022. Questionnaires collected information on socio-demographic characteristics, awareness, knowledge, and practices related to hepatitis B and hepatitis C. Socio-demographic characteristics included age, sex, region, ethnicity, marital status, highest level of education, and employment status. Awareness was defined as having heard of hepatitis B or hepatitis C. Among those who reported being aware of hepatitis B and/or hepatitis C, a series of questions were asked to assess knowledge of transmission and prevention methods for hepatitis B and/or hepatitis C. Survey participants were also asked questions on healthcare practices related to hepatitis B and hepatitis C including ever being tested for hepatitis B, and hepatitis C, and ever being told by a healthcare provider that they have hepatitis B, or hepatitis C. Additionally, questions included information on certain behaviors or practices that might increase the risk of exposure to hepatitis B and hepatitis C such as injection drug use, incarceration, tattooing, piercing, blood transfusion and sharing sharp items. To identify best approaches to disseminate future information on viral hepatitis, we asked participants to list their sources of trusted health information. The survey questionnaire was provided in Russian and Uzbek language and was pilot tested before survey implementation. Majority of the questions had been used in other countries previously [11]– [12].

#### Data analysis

Data were weighted to account for the study design. Two-stage sampling weights were calculated to account for the sampling approach for PHC clinics and survey participants and were adjusted for non-response. Further, post-stratification weights were used to adjust the sampling weights for noncoverage to ensure the survey participants appropriately reflect the distribution of the population of the selected seven regions. Direct calibration was performed based on the population joint distribution within each stratum determined by region, sex, and age group for 2022.

Knowledge scores for hepatitis B and hepatitis C were calculated based on the total number of correct answers. We assigned one point for each correct response to a knowledge question and zero points for incorrect or missing responses. We calculated median knowledge scores with interquartile ranges (IQR). High knowledge was defined as having at least 12 correct answers out of 16 questions for hepatitis B and at least 11 correct answers out of 15 knowledge questions for hepatitis C (i.e. a score of > 70%). Descriptive statistics are presented as weighted proportions and 95% confidence intervals (95% CI). Socio-demographic factors associated with being aware of and having high knowledge for hepatitis B and hepatitis C were assessed through multivariable logistic regression incorporating sampling weights. Adjusted odds ratios (aOR) and 95% CI were computed to assess factors associated with awareness and knowledge. We used linear regression to assess factors associated with knowledge score. Total mean knowledge scores and estimated means difference were computed for each socio-demographic variable. A p-value < 0.05 was considered statistically significant. All analyses were performed using SAS version 9.4 (Cary, North Carolina, USA).

## Results

### Characteristics of the survey population

Of 10,000 targeted adult participants, 9,066 (91%) participated in the survey. Nearly half (46.9%) of participants were younger than 40 years old. While there were almost twice as many female participants (64.2%) compared to males (35.8%) in the survey, data weighting adjusted for that imbalance (Supplemental Table 1). Regional distribution of survey participants was similar to the distribution of the population in Uzbekistan. The majority of survey participants were Uzbek (85.2%), were married or living with a partner (80.6%), and completed university/college or higher education (54.9%). A plurality of survey participants were employed (40.8%).

Given that not all 9,066 survey participants answered the hepatitis B and hepatitis C awareness questions, we presented the socio-demographic characteristics of survey participants who responded to the awareness questions for hepatitis B and hepatitis C separately. The regional distribution of respondents to hepatitis B and hepatitis C awareness was different than the overall 9,066 survey participants (*p* < 0.001). In comparison to the overall sample, respondents to hepatitis B awareness were slightly older, and included more females and persons of non-Uzbek ethnicity (*p* < 0.05). Compared to the total sample, respondents to the hepatitis C awareness question had slightly higher percentages of non-Uzbek, persons with university and higher education, and employed persons (*p* < 0.05) (Supplemental Table 1).

### Awareness of hepatitis B and hepatitis C

Overall, 5,292 persons (58.4%) answered the question on whether they had heard of hepatitis B and weighted population awareness of hepatitis B was 54.7% (95% CI: 46.4–62.8). Hepatitis B awareness was highest among persons aged 40–49 years (60.2%, 95% CI: 49.9–69.8), females (60.5%, 95% CI: 53.2–68.2), persons living in Tashkent city (92.4%, 95% CI: 82.5–96.9), Uzbek (58.9%, 95% CI: 49.8–67.4), persons who were married/living with partner (57.8%, 95% CI: 49.2–65.9), persons with university or higher education (60.8%, 95% CI: 50.7–70.0), and persons who were currently employed (61.3%, 95% CI: 51.8–70.0) (Table 1). After adjusting for all socio-demographic characteristics, age, sex, region, education level and employment status were significantly associated with hepatitis B awareness. Compared to persons aged 18–29 years, those aged 50–59 years had higher odds to be aware of hepatitis B (aOR: 1.86, 95% CI: 1.13–3.05). Women had higher odds to be aware of hepatitis B compared to men (aOR: 2.61, 95% CI: 1.91–3.56). Residents of all regions but Khorezm had significantly lower odds of being aware of hepatitis B compared to Tashkent city. Persons with university education had higher odds of being aware of hepatitis B than those who completed technical/vocational school (aOR: 1.89, 95% CI: 1.32–2.71) (Table 1).

**Table 1:** Awareness of hepatitis B and hepatitis C by demographic characteristics, Uzbekistan, 2022

	Aware of hepatitis B *N* = 5,292				Aware of hepatitis C *N* = 7,925			
	*n*	Weighted % (95% CI)	Adjusted OR^†^ (95% CI)	*P*-value	*n*	Weighted % (95% CI)	Adjusted OR^†^ (95% CI)	*P*-value
**Overall**	2,824	54.7 (46.4–62.8)			3,043	39.0 (33.0–45.4)		
**Age Group (years)**								
18–29	527	48.5 (39.4–57.6)	1		592	36.5 (29.1–44.5)	1	
30–39	816	56.8 (48.4–64.8)	1.43 (0.97–2.11)	0.07	862	39.9 (32.9–47.4)	1.05 (0.82–1.34)	0.69
40–49	588	60.2 (49.9–69.8)	1.39 (0.97–1.99)	0.07	657	44.0 (36.7–51.7)	1.25 (0.95–1.65)	0.11
50–59	507	58.4 (47.2–68.8)	1.86 (1.13–3.05)	0.02	538	42.5 (35.6–49.8)	1.30 (0.85–2.00)	0.22
≥60	386	51.1 (42.0–60.1)	2.06 (0.97–4.37)	0.06	394	32.8 (28.5–37.4)	1.06 (0.63–1.80)	0.81
**Sex**								
Male	749	48.2 (39.1–57.4)	1		832	33.8 (26.7–41.8)	1	
Female	2,075	60.5 (52.3–68.2)	2.61 (1.91–3.56)	< 0.001	2,211	44.2 (38.9–49.7)	2.15 (1.76–2.63)	< 0.001
**Region**								
Tashkent city	798	92.4 (82.5–96.9)	1		792	60.1 (48.7–70.5)	1	
Andijan	289	53.3 (40.7–65.6)	0.14 (0.04–0.42)	< 0.001	317	23.4 (17.0–31.2)	0.21 (0.10–0.42)	< 0.001
Kashkadarya	355	62.3 (43.0–78.4)	0.25 (0.06–0.93)	0.04	378	36.8 (23.5–52.6)	0.71 (0.31–1.62)	0.41
Khorezm	401	83.6 (76.0–89.1)	0.72 (0.22–2.33)	0.57	421	80.2 (72.8–86.0)	4.39 (1.88–10.24)	< 0.001
Karakalpakstan	264	28.9 (18.8–41.5)	0.04 (0.01–0.13)	< 0.001	331	32.8 (26.8–39.4)	0.40 (0.20–0.80)	0.01
Samarkand	230	46.0 (21.4–72.8)	0.10 (0.02–0.43)	0.002	262	19.9 (10.1–35.5)	0.29 (0.11–0.74)	0.01
Tashkent region	487	32.9 (20.9–47.8)	0.05 (0.01–0.15)	< 0.001	542	38.6 (30.4–47.7)	0.48 (0.25–0.92)	0.03
**Ethnicity**								
Uzbek	2,272	58.9 (49.8–67.4)	1		2,444	40.3 (33.4–47.6)	1	
Non-Uzbek	395	36.9 (26.6–48.6)	0.88 (0.56–1.38)	0.58	434	34.4 (27.8–41.7)	0.79 (0.58–1.08)	0.14
**Marital Status**								
Never married	207	44.0 (32.5–56.1)	1		239	31.9 (23.1–42.2)	1	
Married/living with partner	2,214	57.8 (49.2–65.9)	1.49 (0.96–2.31)	0.07	2,366	42.1 (36.2–48.3)	1.33 (0.89–2.01)	0.17
Separated/divorced/widowed	221	51.1 (40.7–61.4)	1.12 (0.67–1.86)	0.67	239	38.5 (30.7–46.9)	1.15 (0.71–1.88)	0.57
**Highest level of education completed**								
Secondary school or less	30	33.3 (17.8–53.3)	0.38 (0.14–1.04)	0.06	36	26.8 (13.1–46.9)	0.64 (0.32–1.29)	0.21
Technical/vocational school	827	49.7 (37.6–61.7)	1		846	29.0 (21.3–38.1)	1	
University or higher	1,785	60.8 (50.7–70.0)	1.89 (1.32–2.71)	< 0.001	1,955	48.4 (41.7–55.1)	2.30 (1.63–3.25)	< 0.001
**Employment Status**								
Employed	1,397	61.3 (51.8–70.0)	1		1,492	46.8 (38.2–55.6)	1	
Retired	336	46.5 (36.7–56.6)	0.40 (0.25–0.63)	< 0.001	355	33.9 (28.9–39.2)	0.52 (0.37–0.74)	< 0.001
Homemaker	648	51.6 (44.0–59.1)	0.58 (0.39–0.86)	< 0.001	677	37.9 (31.4–44.9)	0.56 (0.41–0.75)	< 0.001
Unemployed/student	277	52.6 (34.6–69.9)	1.20 (0.62–2.35)	0.59	326	31.5 (24.1–40.0)	0.71 (0.49–1.02)	0.07

*Abbreviations CI* confidence interval, *OR* odds ratio

^†^ adjusted for all variables in the table

Overall, 7,925 persons (87.4%) answered the question on whether they had heard of hepatitis C. Weighted population awareness of hepatitis C was 39.0% (95% CI: 33–45.4). Hepatitis C awareness was highest among those aged 40–49 years (44.0%, 95% CI: 36.7–51.7), females (44.2%, 95%CI: 38.9–49.7), persons living in Khorezm region (80.2%, 95% CI: 72.8–86.0), Uzbek (40.3%, 95% CI: 33.4–47.6), persons who were married/living with partner (42.1%, 95% CI: 36.2–48.3), persons with university or higher education (48.4%, 95% CI: 41.7–55.1), and persons who were currently employed (46.8%, 95% CI: 38.2–55.6) (Table 1). After adjusting for all socio-demographic characteristics, sex, region, education level and employment status were significantly associated with hepatitis C awareness. Women had higher odds of being aware of hepatitis C than men (aOR: 2.15, 95% CI:1.76–2.63). With the exception of Khorezm and Kashkadarya, residents of all other regions had significantly lower odds of hepatitis C awareness compared to Tashkent city. Persons with university education had higher odds of being aware of hepatitis C than those who finished technical/vocational school (aOR: 2.30, 95% CI: 1.63–3.25) (Table 1).

### Knowledge of hepatitis B and hepatitis C

Among persons who reported being aware of hepatitis B (*n* = 2,824), 2.4% (95% CI: 0.9–6.4) correctly identified all modes of hepatitis B transmission and 2.0% (95% CI: 1.0–3.9) correctly identified all prevention methods for hepatitis B. Among participants who reported being aware of hepatitis C (*n* = 3,043), 3.5% (95% CI: 2.2–5.8) correctly identified all modes of hepatitis C transmission and 2.8% (95% CI: 1.6–4.8) correctly identified all prevention methods for hepatitis C (Table 2). Similar proportion of persons knew that HBV (61.7%, 95% CI: 52.8–70.0) and HCV (61.5%, 95% CI: 54.6–67.9) infection could be asymptomatic and majority knew that hepatitis B (77.7%, 95% CI: 72.2–82.3) and hepatitis C (74.8%, 95% CI: 69.5–79.4) can be treated. The vast majority knew that handshakes (93.6%), sharing food (97.8%), respiratory droplets (97.6%), and touching items in public places (98.0%) would not transmit hepatitis B or hepatitis C. Approximately two thirds knew that hepatitis B or hepatitis C can be transmitted by contaminated blood and just over half knew that sharing needles and syringes might transmit hepatitis B or hepatitis C. Few persons knew that sexual contact could transmit hepatitis B (16.0%, 95% CI: 10.4–23.9) or hepatitis C (15.8%, 95% CI: 10.9–22.4) and fewer knew that hepatitis B can be transmitted from mother to child (7.6%, 95% CI: 4.9–11.5). Overall, 19.1% (95% CI: 14.2–25.2) of participants had high knowledge of hepatitis B and 30.2% (23.0–38.4) had high knowledge for hepatitis C (*p* < 0.001) (Table 2).

**Table 2:** Knowledge of hepatitis B and hepatitis C among survey participants, Uzbekistan, 2022

	Hepatitis B virus (HBV) *N* = 2,824		Hepatitis C virus (HCV) *N* = 3,043		*P*-value†
	*n*	Correct answers Weighted % (95% CI)	*n*	Correct answers Weighted % (95% CI)	
**Is it possible to have HBV/HCV infection but not have symptoms?**	1,756	61.7 (52.8–70.0)	1,841	61.5 (54.6–67.9)	0.95
**Are there medications available to treat HBV/HCV infection?**	2,011	77.7 (72.2–82.3)	2,070	74.8 (69.5–79.4)	0.23
**How do you think HBV/HCV is transmitted?**					
Blood (yes)	1,794	66.5 (56.3–75.3)	1,895	64.9 (55.8–73.0)	0.50
Sharing needles or syringes (yes)	1,300	51.4 (42.2–60.5)	1,401	52.3 (44.0–60.4)	0.82
Sharing household objects like razors and toothbrushes (yes)	986	39.0 (31.4–47.1)	803	28.7 (23.4–34.7)	0.001
Sexual contact (yes)	416	16.0 (10.4–23.9)	424	15.8 (10.9–22.4)	0.90
From mother to child (yes)	178	7.6 (4.9–11.5)	––	n/a	--
Handshake with infected person (no)	2,576	93.6 (90.6–95.7)	2,839	95.6 (93.2–97.2)	0.06
Food (no)	2,688	97.8 (96.0–98.8)	2,883	97.1 (94.9–98.3)	0.24
Droplets (no)	2,693	97.6 (95.6–98.7)	2,891	97.3 (95.1–98.5)	0.51
Touching items in public places (no)	2,708	98.0 (96.2–99.0)	2,909	98.1 (95.7–99.1)	0.94
Don’t know	158	5.9 (3.8–9.3)	198	6.1 (4.5–8.1)	0.93
*Correctly identified all modes of transmission*	38	2.4 (0.9–6.4)	96	3.5 (2.2–5.8)	0.28
**What can you do to help prevent HBV/HCV infection?**					
Avoid unsterile/used medical devices (yes)	1,245	51.0 (41.6–60.3)	1,309	47.7 (39.6–55.9)	0.13
Avoid sharing syringes and needles (yes)	1,224	46.1 (38.4–54.0)	1,530	56.0 (48.8–63.0)	< 0.001
Vaccination †	1,157	46.0 (38.3–53.8)	2,212	75.0 (67.9–81.0)	--
Wash hands thoroughly (no)	2,290	83.5 (76.3–88.8)	2,368	79.2 (70.1–86.0)	0.04
Condoms (yes)	196	8.8 (6.1–12.6)	212	8.6 (6.2–11.8)	0.79
Don’t know	178	5.5 (3.9–7.7)	204	6.3 (4.4–9.0)	0.30
*Correctly identified all prevention methods*	49	2.0 (1.0–3.9)	67	2.8 (1.6–4.8)	0.35
***Achieved a high knowledge score (correctly answered > 70% of questions)***	456	19.1 (14.2–25.2)	854	30.2 (23.0–38.4)	< 0.001

*Abbreviations HBV* hepatitis B virus, *HCV* hepatitis C virus, *n/a *not asked,* CI *confidence interval, *IQR* interquartile range

† p-values compare knowledge of hepatitis B and hepatitis C for each variable

Perfect scores for transmission questions were 9 for HBV and 8 for HCV; perfect score for prevention questions was 5 for each virus. † Correct answer was “Yes” for HBV and “No” for HCV

**Fig. 2:**
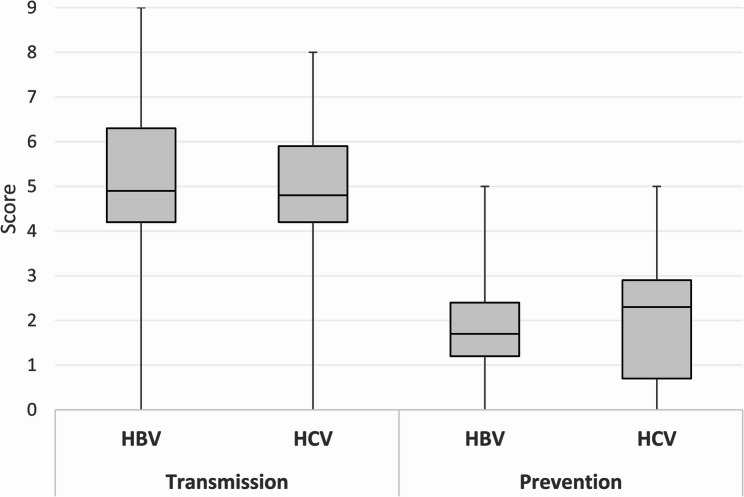
Distribution of hepatitis B and hepatitis C transmission and prevention knowledge scores^†^ among survey participants, Uzbekistan, 2022 Abbreviations: HBV: Hepatitis B virus; HCV: Hepatitis C virus.^† ^Knowledge scores were calculated based on the total number of correct answers. We assigned one point for each correct response to a knowledge question and zero points for incorrect or missing responses. Perfect scores for transmission questions were 9 for HBV and 8 for HCV. Perfect score for prevention questions was 5 for each virus. The line inside the box represents the median and the length of the box represents the inter-quartile range

The median knowledge scores for hepatitis B and hepatitis C transmission were 4.9 (IQR: 4.2–6.3) out of nine and 4.8 (IQR: 4.2–5.9) out of eight, respectively. Median knowledge scores for prevention methods of hepatitis B and hepatitis C were 1.7 (IQR: 1.2–2.4) out of five and 2.3 (IQR: 0.7–2.9) out of five, respectively (Fig. 2). Being 50 years and older, residing in Andijan region, and having secondary or lower education were associated with having lower knowledge scores for hepatitis B compared to those aged 18–29 years, from Tashkent city, and having completed technical/vocational school (Table 3). Compared to those living in Tashkent city, persons living in all other regions but Khorezm and Samarkand had significantly lower knowledge scores for hepatitis C. Persons who were unemployed or were a student had significantly lower knowledge scores for hepatitis C than those who were employed.

**Table 3:** Socio-demographic characteristics associated with hepatitis B and hepatitis C knowledge scores among survey participants, Uzbekistan, 2022

	Hepatitis B			Hepatitis C		
	Mean total knowledge score^†^	Adjusted linear regression^‡^		Mean total knowledge score^§^	Adjusted linear regression^‡^	
		Estimated means difference (95% CI)	*P*-value		Estimated means difference (95% CI)	*P*-value
**Age Group (years)**						
18–29	8.9	1		8.8	1	
30–39	9.1	−0.27 (−0.77, 0.23)	0.280	9.0	−0.12 (−0.61, 0.37)	0.626
40–49	8.4	−0.50 (−1.21, 0.20)	0.161	8.9	−0.12 (−0.56, 0.32)	0.593
50–59	8.5	−0.71 (−1.26, −0.16)	0.012	8.8	−0.26 (−1.00, 0.48)	0.482
≥60	8.3	−1.33 (−2.21, −0.45)	0.004	8.6	−0.57 (−1.25, 0.12)	0.106
**Sex**						
Male	8.8	1		8.6	1	
Female	8.6	−0.29 (−0.73, 0.16)	0.205	9.1	0.25 (−0.11, 0.60)	0.169
**Region**						
Tashkent city	6.6	1		10.1	1	
Andijan	9.7	−2.15 (−3.34, −0.96)	0.001	7.7	−2.13 (−3.80, −0.45)	0.013
Kashkadarya	7.7	−1.42 (−2.96, 0.11)	0.069	7.3	−2.29 (−3.46, −1.11)	< 0.001
Khorezm	7.8	−0.27 (−1.42, 0.89)	0.649	9.4	−0.56 (−1.68, 0.57)	0.328
Karakalpakstan	9.3	−0.58 (−1.80, 0.64)	0.347	8.6	−1.49 (−2.65, −0.34)	0.012
Samarkand	9.3	−2.24 (−4.49, 0.00)	0.050	8.7	−1.10 (−2.83, 0.62)	0.206
Tashkent region	9.1	−0.66 (−2.47, 1.14)	0.469	8.9	−1.28 (−2.52, −0.04)	0.044
**Marital Status**						
Never married	8.6	1		9.0	1	
Married/living with partner	8.9	0.29 (−0.57, 1.14)	0.504	9.0	−0.05 (−0.70, 0.61)	0.888
Separated/divorced/widowed	8.9	0.50 (−0.43, 1.43)	0.291	8.7	−0.04 (−0.85, 0.77)	0.926
**Highest level of education**						
Secondary school or less	7.1	−2.22 (−4.06, −0.38)	0.019	8.3	−0.67 (−1.78, 0.44)	0.235
Technical/vocational school	8.2	1		8.5	1	
University or higher	9.2	0.22 (−0.28, 0.72)	0.392	9.2	0.31 (−0.07, 0.68)	0.105
**Employment Status**						
Employed	9.3	1		9.3	1	
Retired	8.6	−0.06 (−0.75, 0.63)	0.864	8.6	−0.35 (−0.89, 0.19)	0.207
Homemaker	8.9	−0.12 (−0.56, 0.33)	0.603	8.9	−0.24 (−0.64, 0.16)	0.240
Unemployed/Student	7.4	−0.91 (−1.87, 0.05)	0.064	8.0	−0.79 (−1.32, −0.26)	0.004

*Abbreviations* CI confidence interval

^†^ Knowledge score for hepatitis B ranged from 0 to 16

^‡^adjusted for all variables listed in the table

^§^ Knowledge score for hepatitis C ranged from 0 to 15

We used logistic regression to identify socio-demographic factors associated with having high knowledge scores (correctly answered > 70% of questions) for hepatitis B and hepatitis C (Table 4). After adjusting for all characteristics, persons aged 50–59 years (aOR: 0.56; 95% CI: 0.33–0.95) and persons aged ≥ 60 years (aOR: 0.47; 95% CI: 0.24–0.90) had lower odds of having high knowledge for hepatitis B compared to those aged 18–29 years. In addition, persons living in Kashkadarya region had 80% lower odds of having high hepatitis B knowledge (aOR: 0.20; 95% CI: 0.05–0.77) compared to those residing in Tashkent city. For hepatitis C, persons living in Andijan region (aOR: 0.17; 95% CI: 0.06–0.50) and Kashkadarya region (aOR: 0.05; 95% CI: 0.01–0.32) had lower odds of having high hepatitis C knowledge compared to those living in Tashkent city. No other socio-demographic factors were significantly associated with having high hepatitis B or hepatitis C knowledge (Table 4).

**Table 4:** Multivariable analysis of socio-demographic factors associated with having high knowledge† for hepatitis B and hepatitis C, Uzbekistan, 2022

	Hepatitis B Knowledge *N* = 2,824			Hepatitis C Knowledge *N* = 3,034		
Variable	High score Weighted % (95% CI)	Adjusted OR (95% CI)	*P*-value	High score Weighted % (95% CI)	Adjusted OR (95% CI)	*P*-value
**Age, years**						
18–29	21.8 (14.9–30.8)	1		29.5 (20.9–39.8)	1	
30–39	22.0 (15.7–29.8)	0.90 (0.57, 1.41)	0.63	32.6 (24.3–42.2)	0.92 (0.60, 1.42)	0.72
40–49	16.2 (10.9–23.4)	0.64 (0.38, 1.06)	0.08	28.9 (20.2–39.4)	0.74 (0.47, 1.15)	0.17
50–59	16.1 (10.7–23.5)	0.56 (0.33, 0.95)	0.03	31.0 (22.4–41.2)	0.83 (0.50, 1.37)	0.47
≥60	17.0 (11.4–24.6)	0.47 (0.24, 0.90)	0.02	27.4 (18.7–38.1)	0.58 (0.29, 1.19)	0.13
**Sex**						
Male	18.3 (13.2–24.6)	1		28.4 (20.4–37.9)	1	
Female	19.7 (14.1–27.0)	1.00 (0.70, 1.43)	1.00	31.5 (24.1–40.1)	1.00 (0.72, 1.39)	0.99
**Region**						
Tashkent city	24.5 (15.4–36.6)	1		50.2 (28.7–71.6)	1	
Andijan	15.2 (5.6–34.8)	0.43 (0.16, 1.18)	0.10	15.6 (8.3–27.2)	0.17 (0.06, 0.50)	0.002
Kashkadarya	5.3 (1.7–15.4)	0.20 (0.05, 0.77)	0.02	4.6 (0.9–20.2)	0.05 (0.01, 0.32)	0.002
Khorezm	24.7 (21.3–28.4)	1.12 (0.64, 1.97)	0.68	33.5 (28.3–39.2)	0.51 (0.20, 1.30)	0.16
Karakalpakstan	13.3 (5.0–30.9)	0.43 (0.12, 1.54)	0.19	32.0 (22.0–43.9)	0.43 (0.15, 1.23)	0.11
Samarkand	14.2 (3.7–41.5)	0.66 (0.17, 2.55)	0.55	24.5 (10.7–46.9)	0.33 (0.08, 1.33)	0.12
Tashkent region	27.0 (12.4–49.0)	1.11 (0.38, 3.25)	0.84	30.6 (18.6–46.0)	0.39 (0.13, 1.18)	0.09
**Marital status**						
Never married	20.0 (12.3–30.7)	1		30.1 (19.2–43.8)	1	
Married/living with partner	20.3 (15.2–26.5)	1.05 (0.59, 1.88)	0.87	32.2 (24.9–40.5)	1.12 (0.73, 1.72)	0.59
Separated/divorced/widowed	19.9 (12.0–31.2)	0.99 (0.48, 2.03)	0.98	28.9 (18.8–41.5)	1.15 (0.63, 2.09)	0.65
**Highest level of education completed**						
Secondary school or less	5.9 (1.4–21.3)	0.20 (0.03, 1.28)	0.09	13.6 (4.1–36.9)	0.35 (0.06, 2.05)	0.24
Technical/vocational school	16.8 (10.9–24.8)	1		25.8 (17.8–35.7)	1	
University or higher	21.6 (15.7–29.0)	1.11 (0.74, 1.67)	0.61	33.7 (24.9–43.7)	1.05 (0.65, 1.68)	0.85
**Employment status**						
Employed	22.4 (16.5–29.6)	1		36.7 (27.9–46.5)	1	
Retired	17.6 (11.8–25.5)	1.04 (0.53, 2.03)	0.92	28.6 (19.8–39.5)	0.88 (0.45, 1.74)	0.72
Homemaker	19.2 (13.3–27.1)	0.84 (0.52, 1.36)	0.48	28.4 (20.8–37.4)	0.79 (0.52, 1.19)	0.25
Unemployed/Student	13.7 (6.7–26.1)	0.79 (0.40, 1.54)	0.48	19.6 (11.5–31.3)	0.62 (0.39, 1.00)	0.05

*Abbreviations* *CI *confidence interval, *OR* odds ratio

† High knowledge score was defined as having at least 12 correct answers out of 16 questions for hepatitis B and at least 11 correct answers out of 15 knowledge questions for hepatitis C (> 70% correct answers to transmission and prevention methods)

### Testing and behavioral practices related to hepatitis B and hepatitis C

Table 5 presents weighted percentages for testing practices and behaviors related to hepatitis B and hepatitis C that were self-reported by study respondents. Among survey participants who answered the questions, 28.8% (95% CI: 18.1–42.6) reported having ever been tested for hepatitis B and 8.2% (95% CI: 5.6–12.0) reported having been told by a healthcare provider that they have hepatitis B. In comparison, 17.7% (95% CI: 12.9–23.8) reported having ever been tested for hepatitis C and 2.3% (95% CI: 1.5–3.6) were told by a healthcare provider that they have hepatitis C. Awareness of hepatitis B among respondents who reported having been told by a healthcare provider to have hepatitis B was 84.4% (95% CI: 69.1–92.9) compared to 60.5% (95% CI: 39.6–78.2) among those who reported not having hepatitis B (*p* = 0.07). Among those aware of hepatitis B, 11.7% (95% CI: 5.4–23.4) of those who reported being diagnosed with hepatitis B had high knowledge, compared to 16.4% (95% CI: 9-7−26.4) of those who had not been diagnosed with hepatitis B (*p* = 0.42). In comparison, survey participants who reported being told to have hepatitis C had significantly higher awareness of hepatitis C (86%, 95%CI:71.9–93.7) than those who reported not being diagnosed with hepatitis C (37.7%, 95% CI: 31.6–44.3) (*p* < 0.001). Among those aware of hepatitis C, 29.4% (95% CI: 16.7–46.5) of those who reported being diagnosed with hepatitis C had high knowledge, compared to 32.2% (95% CI: 24.4–41.2) of those who had not been diagnosed with hepatitis C (*p* = 0.71).

**Table 5:** Hepatitis B and hepatitis C testing and behaviors that might increase the risk of exposure to hepatitis B and hepatitis C among survey participants, Uzbekistan, 2022

	*n*	Weighted % (95% CI)
**Testing and clinical care**		
Ever tested for hepatitis B (*N* = 2069)	659	28.8 (18.1–42.6)
Told by healthcare provider they have hepatitis B (*N* = 1722)	132	8.2 (5.6–12.0)
Ever tested for hepatitis C (*N* = 7815)	1,453	17.7 (12.9–23.8)
Told by healthcare provider they have hepatitis C (*N* = 7362)	175	2.3 (1.5–3.6)
**Behaviors**		
Ever been incarcerated (yes) (*N* = 7513)	66	1.2 (0.6–2.3)
Ever injected drugs (yes) (*N* = 7361)	37	0.4 (0.1–1.8)
Ever used someone else’s needle after they used it (yes) (*N* = 24)	22	97.4 (3.9–100.0)
Ever let someone else use their needle after they have used it (yes) (*N* = 24)	20	93.4 (1.4–100.0)
Has tattoos (yes) (*N* = 7074)	182	2.6 (1.7–3.8)
Place of tattoo(s) receipt (*N* = 172)		
Beauty salon or tattoo salon	97	43.1 (25.2–63.1)
Home	21	12.0 (5.9–22.8)
Army	28	16.8 (8.3–31.2)
Prison	6	9.5 (3.7–22.1)
Other	18	18.0 (7.3–37.9)
Don’t know/remember	2	0.6 (0.1–3.3)
Type of needles used for their tattoo(s) (*N* = 169)		
New	136	76.7 (62.4–86.6)
Used	9	7.8 (2.5–21.8)
Don’t know/remember	24	15.5 (7.8–28.5)
Used or new bottle of ink was used for their tattoo (s) (*N* = 165)		
New	116	71.7 (57.7–82.5)
Used	12	9.9 (3.7–24.1)
Don’t know/remember	37	18.4 (11.0–29.1)
Has piercings (yes) (*N* = 6056)	1,198	15.5 (9.9–23.5)
Place where they got their piercings (*N* = 1175)		
Beauty salon or tattoo salon	128	12.7 (6.1–24.6)
Home	961	79.3 (70.1–86.2)
Prison	1	0.1 (0.0–0.6)
Elsewhere	5	0.2 (0.0–0.7)
Don’t remember	81	7.8 (3.5–16.5)
Ever shared the following items with family members (yes) (*N* = 6566)		
Toothbrushes	80	1.2 (0.5–2.5)
Razor blades (for shaving)	85	1.7 (0.8–3.7)
Scissors used for grooming	1,119	19.1 (9.9–33.8)
Shaving brush	112	2.6 (0.8–7.9)
Nail cutter	100	1.3 (0.7–2.3)
I don’t share any of these items	4,502	68.6 (55.8–79.1)

The denominator was different for each variable due to missing responses and skipping patterns incorporated in the questionnaire based on previous responses

*Abbreviations CI *confidence interval

In terms of potential behaviors that might increase the risk of exposure to HBV or HCV infection, 1.2% (95% CI: 0.6–2.3) had ever been incarcerated, 0.4% (95% CI: 0.1–1.8) reported ever injecting drugs, 2.6% (95% CI: 1.7–3.8) had tattoos, and 15.5% (95% CI: 9.9–23.5) had piercings. Among the small number of participants (*n* = 37) who reported ever injecting drugs, 97.4% (95% CI: 3.9–100.0) reported ever using someone else’s needle. Among those who had a tattoo, 16.8% (95% CI: 8.3–31.2) reported getting a tattoo while in the army, 9.5% (95% CI: 3.7–22.1) reported getting the tattoo in prison, and 7.8% (95% CI: 2.5–21.8) reported that previously-used needles were used by the person who gave them the tattoo. Of those who had a piercing, 79.3% (95% CI: 70.1–86.2) had their piercing done at home.

In this surveyed population, 15.9% (95% CI: 12.1–20.8) reported ever donating blood in the past, while 5.0% (95% CI: 3.4–7.1) reported ever receiving a blood transfusion. Among those who previously donated blood, 42.3% (95% CI: 27.1–59.2) donated blood to family and friends and 8.5% (95% CI: 3.1–21.4) donated blood in exchange for money.

**Fig. 3:**
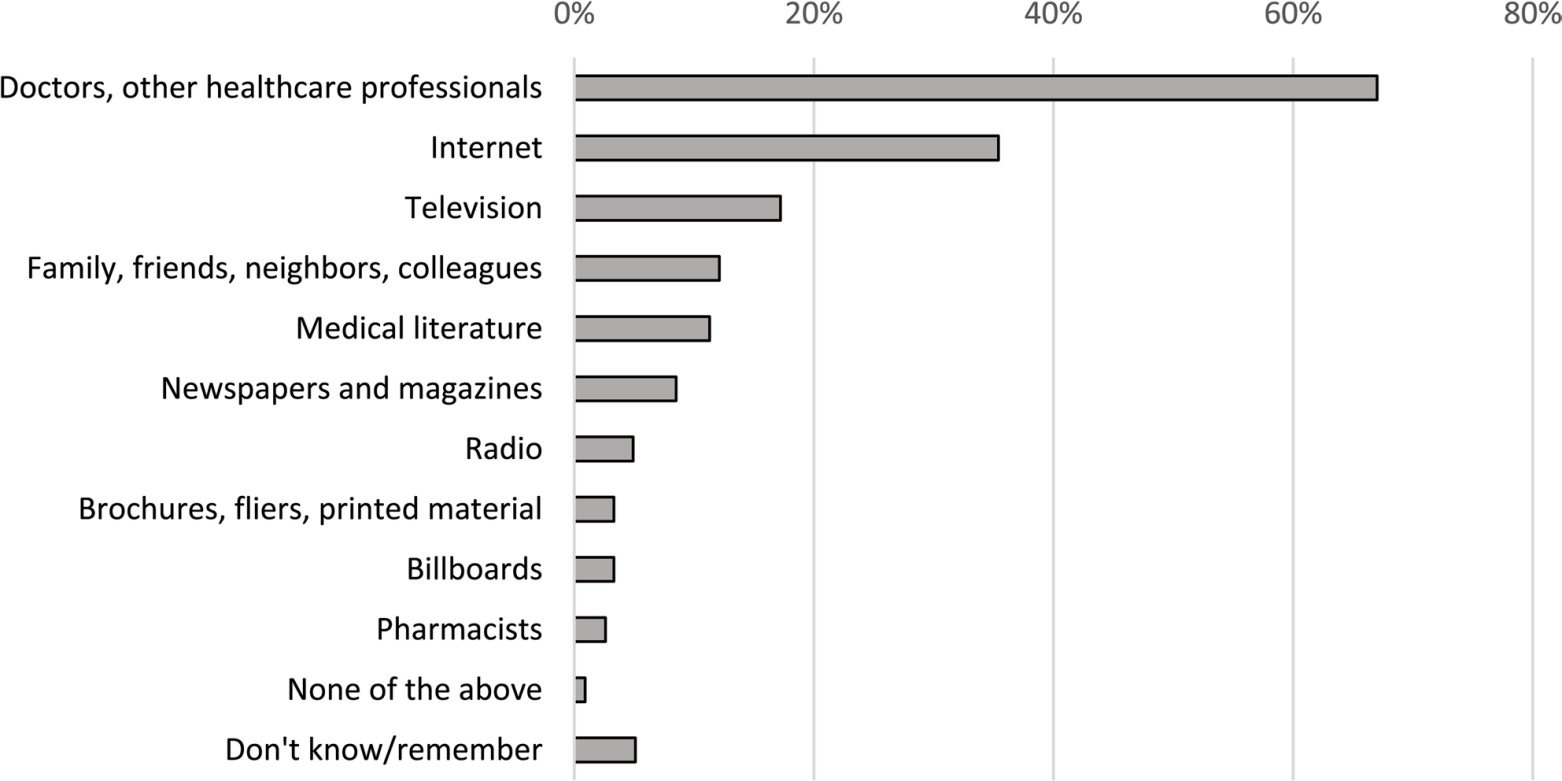
Sources of trusted health-related information among survey participants†, Uzbekistan 2022 (*N* = 5,345) † Answers are not mutually exclusive. Respondents can select multiple sources

Overall, 5,345 respondents reported being interested in health information. The most trusted sources of health information were healthcare professionals (67.0%; 95% CI: 58.5–74.6), internet (35.4%; 95% CI: 26.8–45.0), and television (17.2%; 95% CI: 11.2–25.5) (Fig. 3). Healthcare professionals were the most trusted source of information across all age groups, sex, regions, and education levels. The internet and television were the next two most trusted sources of health information across all socio-demographic characteristics. However, their ranking as either second or third preferred source of health information varied region. More males mentioned the internet as a trusted source of health information compared to females (41.5% vs. 29.7%; *p* < 0.001). Participants aged 18 – 29 years had higher trust in seeking health information on the internet compared to those aged ≥ 60 years (43.4% vs. 19.3%; *p* < 0.001). We did not observe other differences in trusted sources of health information by age or sex. Respondents having a university or higher education level less frequently reported trusting information from pharmacists (*p* < 0.001), newspapers (*p* = 0.04), and billboards (*p* = 0.04) compared to those with secondary or lower education levels.

## Discussion

This is the first survey to assess awareness, knowledge, and practices related to viral hepatitis in the general population in Uzbekistan. Results show that more than half of the population were aware of hepatitis B and 39.0% have previously heard of hepatitis C. Knowledge of transmission and prevention methods was low among those who reported having previously heard of hepatitis B or hepatitis C. In addition, significant gaps were identified in practices and knowledge of prevention and transmission methods for hepatitis B and hepatitis C.

Awareness was higher for hepatitis B (55%) than hepatitis C (39%) which could be related to the higher prevalence of hepatitis B compared to hepatitis C in the population in Uzbekistan [7]. A study across 11 countries and territories in Asia reported a correlation between hepatitis B and hepatitis C prevalence and death rates with awareness and knowledge levels of hepatitis B and hepatitis C [13]. Countries with higher prevalence had higher awareness and knowledge of hepatitis B or hepatitis C. In addition, hepatitis B has been recognized as a separate infection since the 1960 s and gradual introduction of hepatitis B vaccine during 1998–2001 in former Soviet Union countries also likely contributed to higher awareness of hepatitis B [8, 14]. On the other hand, hepatitis C was defined as non-A non-B hepatitis for several years, with no studies assessing its burden in former Soviet Union countries until early 2000 s, which might have impacted hepatitis C awareness in the population [15]– [16]. The overall awareness of hepatitis B in Uzbekistan was similar to other countries in South-East Asia but higher than reported awareness in the general population in the country of Georgia (35%) [11, 17]. However, population awareness of hepatitis C in Uzbekistan was almost half the awareness reported in Georgia (66%) [12]. Unlike Uzbekistan, Georgia implemented broad awareness and educational campaigns as they launched their hepatitis C elimination program in 2015 [18]. As Uzbekistan implements its resolution to expand prevention, testing, and treatment of hepatitis B and hepatitis C, it would be essential to conduct nationwide awareness campaigns on hepatitis B and hepatitis C in the general population to increase testing for hepatitis B and hepatitis C and prevent new infections. Awareness campaigns could be disseminated by primary healthcare providers, and on social media and television networks as these were the most trusted sources of health information in this population.

Consistent with studies in other countries, awareness and knowledge of hepatitis B and hepatitis C in Uzbekistan were associated with socio-economic status including living in a large city, higher education, and being employed [13, 17, 19–23]. People with higher education have been shown to have higher knowledge of hepatitis B or hepatitis C in several countries [19–23]. Therefore, to reach people with lower educational levels, health awareness sessions need to be tailored to be understood by lay persons irrespective of their education level. Strategies to reach people with lower education could involve community-based sessions using stories and active involvement in learning [24, 25]. In addition, women had higher awareness of both hepatitis B and hepatitis C compared to men. However, no statistically significant association was found between knowledge and sex in the multivariable analysis. Surveys in Greece and France reported significantly higher odds of having high hepatitis B and/or hepatitis C knowledge among women compared with men [19, 26]. However, surveys in Ethiopia showed that the odds of having high knowledge were higher among men compared with women [27]. Persons older than 50 years had lower odds of having high knowledge of hepatitis B compared to younger age groups, which could be related to younger people in Uzbekistan having better access to information through social media and internet. Surveys in Greece, Australia and Canada reported similar trends where older age was associated with lower odds of having a high knowledge of viral hepatitis [19, 21, 23]. Given the variability in awareness and knowledge based on socio-demographic characteristics, tailored educational programs would be most effective at reaching people with various socio-demographic characteristics. Systematic reviews have shown that tailored interventions based on specific audience characteristics were more effective at improving health outcomes compared to non-tailored educational messages [24, 25, 28].

The survey population in Uzbekistan did not have a high prevalence of misconceptions related to hepatitis B and hepatitis C transmission (handshake, food, droplets, touching items) seen in other studies [13]. However, knowledge of actual risk factors was low overall; only two thirds identified blood as a source of transmission of hepatitis B and hepatitis C, half knew that sharing syringes and needles is a risk factor, < 20% identified risk from sexual contact, and < 10% reported that hepatitis B could be transmitted from mother to child. These proportions are lower than reported knowledge of risk factors for hepatitis B and hepatitis C in Australia, Georgia, Greece, and Vietnam [11, 12, 19, 21, 29], but similar to proportions reported in Ethiopia [27]. This low knowledge of risk factors was also reflected in the low knowledge of prevention methods whereby < 10% mentioned use of condoms to prevent HBV or HCV infection. Low knowledge of risk factors might have influenced engagement in practices that might increase the risk of infection, such as sharing needles and syringes (among the few respondents who reported injection drug use) and getting tattoos in prisons and in the army. Incorporating behavioral change interventions whereby behavior change themes are included in mass awareness campaigns, in addition to sharing knowledge about viral hepatitis, might encourage people to implement prevention measures and also seek testing for hepatitis B and hepatitis C [24, 25, 28].

This survey covered almost 60% of the population of Uzbekistan and is one of the largest surveys to assess awareness and knowledge of hepatitis B and hepatitis C in the general population. However, limitations include recall bias and social desirability bias due to the self-reported information. In addition, we could not measure associations between knowledge and practices due to the small numbers of participants reporting engagement in certain behaviors or practices. Use of logistic regression in a cross-sectional survey may have overestimated the effect sizes due to the high frequency of awareness and high knowledge. Due to the use of paper-based questionnaires, we could not ensure that all required questions had an answer, leading to fewer participants responding to certain questions. A substantial proportion of survey participants did not have responses for hepatitis B and hepatitis C awareness, the reasons for which could not be determined. Differences in some socio-demographic characteristics between the total survey respondents and respondents to hepatitis B and hepatitis C awareness questions might impact the generalizability of results. However, the sample size was large enough to provide estimates on hepatitis B and hepatitis C awareness and knowledge in those seven regions. While the questionnaire was pilot tested and provided in local languages, there is no validated viral hepatitis knowledge, attitude and practices survey questionnaire that is available for use. Finally, due to cultural sensitivities, questions on sexual practices could not be included in the survey, which limits the ability to assess these behaviors in the population.

## Conclusions

This survey found low awareness and knowledge of, and limited screening for, hepatitis B and hepatitis C in Uzbekistan. Socio-demographic factors were associated with awareness and knowledge levels. Healthcare professionals and the internet were the most trusted sources of health information in the population. Hence, educational campaigns involving healthcare professionals and dissemination of information through social media are needed to promote population awareness and address knowledge gaps in order to increase uptake of interventions aimed at eliminating viral hepatitis in Uzbekistan.

## Supplementary Information

Supplementary Material 1.

## Data Availability

The data that support the findings of this study are available on request from the Ministry of Health of Uzbekistan. The data are not publicly available due to privacy or ethical restrictions.

## Abbreviations

aOR: Adjusted odds ratios
CDC: Centers for disease control and prevention
CI: Confidence intervals
HBV: Hepatitis B virus
HCV: Hepatitis C virus
IQR: Interquartile range
PHC: Primary health care
