# Supplementary material for: Awareness, knowledge, and practices related to hepatitis B and hepatitis C in the Republic of Uzbekistan—Results from a population-based survey, 2022
Source: BMC Public Health. 2026 Jan 23;26:628. doi: 10.1186/s12889-025-25990-1 (PMC12911132; doi:10.1186/s12889-025-25990-1)
Supplement: Supplementary file 1 — Supplementary Material 1. [file 12889_2025_25990_MOESM1_ESM.docx]

**Supplemental Table 1. Socio-demographic characteristics of survey participants, Uzbekistan, 2022**

|  | **Total** **(N = 9,066)** | | **Responded to hepatitis B awareness  (N = 5,292)** | | **Responded to hepatitis C awareness**  **(N = 7,925)** | |
| --- | --- | --- | --- | --- | --- | --- |
|  |  |  |  |  |  |  |
|  | **n**  **(crude %)** | **Weighted % (95%CI)** | **n**  **(crude %)** | **Weighted % (95%CI)** | **n**  **(crude %)** | **Weighted % (95%CI)** |
| **Age Group (years)** |  |  |  |  |  |  |
| 18-29 | 1,898 (20.9) | 29.1 (26.8–31.5) | 1,091 (20.6) | 27.0 (24.4–29.7) | 1,645 (20.8) | 28.9 (26.7–31.3) |
| 30-39 | 2,358 (26.0) | 25.3 (23.6–27.2) | 1,407 (26.6) | 25.1 (22.8–27.6) | 2,052 (25.9) | 25.8 (24.4–27.1) |
| 40-49 | 1,775 (19.6) | 18.0 (16.7–19.4) | 1,068 (20.2) | 20.0 (17.9–22.2) | 1,535 (19.4) | 17.9 (16.5–19.4) |
| 50-59 | 1,542 (17.0) | 13.8 (12.4–15.4) | 916 (17.3) | 14.9 (13.2–16.7) | 1,370 (17.3) | 13.6 (12.2–15.1) |
| ≥60 | 1,493 (16.5) | 13.8 (12.7–14.9) | 810 (15.3) | 13.1 (11.9–14.4) | 1,323 (16.7) | 13.8 (12.7–15.0) |
| **Sex** |  |  |  |  |  |  |
| Male | 3,245 (35.8) | 49.4 (46.1–52.7) | 1,799 (34.0) | 47.0 (44.0–50.0) | 2,812 (35.5) | 49.9 (47.1–52.7) |
| Female | 5,821 (64.2) | 50.6 (47.3–53.9) | 3,493 (66.0) | 53.0 (50.0–56.0) | 5,113 (64.5) | 50.1 (47.3–52.9) |
| **Region** |  |  |  |  |  |  |
| Andijan | 1,555 (17.2) | 15.8 (8.7–27.1) | 589 (11.1) | 10.3 (5.4–18.7) | 1,400 (17.7) | 16.2 (8.6–28.2) |
| Kashkadarya | 1,463 (16.1) | 16.1 (10.9–23.2) | 726 (13.7) | 13.1 (8.8–19.0) | 1,369 (17.3) | 17.4 (11.7–25.1) |
| Khorezm | 844 (9.3) | 9.5 (5.9–14.7) | 462 (8.7) | 9.0 (4.6–16.8) | 540 (6.8) | 7.0 (3.6–13.2) |
| Karakalpakstan | 911 (10.0) | 9.6 (5.2–17.1) | 757 (14.3) | 13.9 (6.6–26.7) | 903 (11.4) | 11.0 (5.9–19.6) |
| Samarkand | 1,546 (17.1) | 19.2 (11.8–29.6) | 585 (11.1) | 13.1 (7.8–21.2) | 1,075 (13.6) | 14.9 (7.5–27.3) |
| Tashkent city | 1,279 (14.1) | 14.9 (11.5–19.2) | 859 (16.2) | 17.1 (12.4–23.2) | 1,275 (16.1) | 17.2 (13.1–22.3) |
| Tashkent region | 1,468 (16.2) | 14.8 (10.3–21.0) | 1,314 (24.8) | 23.6 (16.4–32.7) | 1,363 (17.2) | 16.3 (11.1–23.4) |
| **Ethnicity** |  |  |  |  |  |  |
| Uzbek | 7,091 (85.2) | 87.3 (81.3–91.6) | 4,008 (80.6) | 82.8 (73.6–89.3) | 6,254 (84.2) | 86.1 (79.4–90.9) |
| Non-Uzbek | 1,231 (14.8) | 12.7 (8.4–18.7) | 962 (19.4) | 17.2 (10.7–26.4) | 1,175 (15.8) | 13.9 (9.1–20.6) |
| **Marital Status** |  |  |  |  |  |  |
| Never married | 866 (10.7) | 15.4 (13.6–17.3) | 494 (10.1) | 14.0 (12.4–15.8) | 768 (10.6) | 15.8 (14.1–17.6) |
| Married/living with partner | 6,503 (80.6) | 77.1 (75.2–79.0) | 3,958 (81.3) | 78.7 (76.1–81.1) | 5,842 (80.9) | 77.2 (75.2–79.2) |
| Separated/divorced/widowed | 701 (8.7) | 7.5 (5.7–9.8) | 418 (8.6) | 7.3 (5.5–9.7) | 608 (8.4) | 7.0 (5.2–9.4) |
| **Highest level of education completed** | | | | | | |
| Secondary school or less | 218 (2.8) | 2.7 (1.9–3.9) | 112 (2.3) | 2.3 (1.4–3.7) | 174 (2.4) | 2.4 (1.6–3.5) |
| Technical/vocational school | 3,320 (42.3) | 42.8 (36.0–49.8) | 1,842 (38.0) | 38.4 (32.4–44.8) | 2,948 (41.1) | 39.9 (33.1–47.2) |
| University or higher | 4,306 (54.9) | 54.5 (47.4–61.4) | 2,896 (59.7) | 59.3 (52.7–65.6) | 4,043 (56.4) | 57.7 (50.4–64.7) |
| **Employment Status** |  |  |  |  |  |  |
| Employed | 3,289 (40.8) | 43.0 (38.3–47.7) | 2,231 (45.2) | 46.3 (40.8–51.9) | 3,027 (41.4) | 44.5 (40.2–48.9) |
| Retired | 1,345 (16.7) | 13.2 (11.9–14.6) | 753 (15.3) | 12.5 (10.8–14.3) | 1,247 (17.1) | 13.4 (12.0–14.9) |
| Homemaker | 2,145 (26.6) | 21.4 (18.6–24.4) | 1,248 (25.3) | 20.7 (17.6–24.1) | 1,899 (26.0) | 20.8 (17.9–23.9) |
| Unemployed/student | 1,275 (15.8) | 22.5 (18.4–27.1) | 701 (14.2) | 20.6 (15.4–26.9) | 1,132 (15.5) | 21.3 (17.4–25.8) |
